# The Discovery of Aurora Kinase Inhibitor by Multi-Docking-Based Virtual Screening

**DOI:** 10.3390/ijms151120403

**Published:** 2014-11-06

**Authors:** Jun-Tae Kim, Seo Hee Jung, Sun Young Kang, Chung-Kyu Ryu, Nam Sook Kang

**Affiliations:** 1 Graduate School of New Drug Discovery and Development, Chungnam National University, Daehakno 99, Yuseong-gu, Daejeon 305-764, Korea; E-Mails: wowjtkim@gmail.com (J.-T.K.) seohee1114@naver.com (S.H.J.); 2 AccelrysKorea, Korea BioPark Bldg C-dong 602, Sampyeong-dong, Bundang-gu, Seongnami-si, Gyeonggi-do 463-400, Korea; E-Mail: neons20@hanmail.net; 3 College of Pharmacy & Graduate School of Pharmaceutical Sciences, Ewha Womans University, 52, Ewhayeodae-gil, Seodaemun-gu, Seoul 120-75, Korea; E-Mail: ckryu@ewha.ac.kr

**Keywords:** aurora kinase, FBDD, docking, cancer, virtual screening

## Abstract

We report the discovery of aurora kinase inhibitor using the fragment-based virtual screening by multi-docking strategy. Among a number of fragments collected from eMololecules, we found four fragment molecules showing potent activity (>50% at 100 μM) against aurora kinase. Based on the explored fragment scaffold, we selected two compounds in our synthesized library and validated the biological activity against Aurora kinase.

## 1. Introduction

The aurora kinase, which belongs to the group of serine/threonine kinases, has been identified as a crucial regulator of the centrosome function in mitosis [1]. In mammals, the aurora family consists of three kinase members, known as aurora-A, -B, and -C, respectively. All aurora kinases share nearly 70% sequence homology among family members [2]. Despite these high similarities, aurora kinases are clearly distinguishable by means of subcellular localization, their expression patterns, and the timing of their activity. Aurora-A is localized to centrosomes during the early S phase and is essential for centrosome maturation and separation, bipolar spindle assembly, and mitotic entry and exit [3]. Aurora-A is frequently overexpressed in many human tumors, including those of breast, ovarian, lung, and colorectal cancers [2]. Aurora-A plays a critical role in the cell cycle and in carcinogenesis, and it has been studied as an anticancer therapeutic target by many researchers. Various aurora-A kinase inhibitors have been reported to have undergone Phase I/II clinical trials to target certain types of cancers. For instance, **CYC116**, a type of pyrimidine analogue, is an orally available aurora kinase inhibitor that is currently undergoing Phase I clinical trials [4]. **MLN8054**, a type of benzopyrimidoazepine analogue, is a potent and selective aurora-A inhibitor with an half maximal inhibitory concentration of a substance (IC_50_) value of 4 nM: It is also under Phase I research for malignant tumors [5].

Our goal was to discover a potent fragment to serve as an aurora-A kinase inhibitor leading to the development of a preclinical drug. To find a hit compound, the typical high-throughput screening (HTS) method from huge chemical library having full-size molecules, *i.e.*, 400~500 Dalton of molecular weight, is carried out. However, this typical HTS method is too expensive in terms of time and energy efficiency [6]. We ruminated on a low-cost and highly effective approach with high reliability criteria to overcome the disadvantages of the typical HTS method. According to the literature, a fragment has the low affinity for proteins, but typically a good ligand efficiency that represents high-quality interactions with its target protein [7]. Since it is well known that fragment screening is efficient in the early stages of drug discovery, we applied the fragment-based virtual high-throughput screening (vHTS) approach to achieve the aforementioned advantages and carried out a docking experiment with a fragment library into the active site of the aurora-A kinase via the X-ray crystallography method.

## 2. Results and Discussion

### 2.1. The Selection of Compounds

Initially, we selected 1,363,325 compounds from a very large database, and 5000 diverse fragments among them were selected as representative molecules according to the processes described in the experimental section.

From the 5000 diverse fragments selected, we carried out a docking study in two steps. To determine the key interaction site and to consider the protein’s flexibility for the docking study, we analyzed the structural characteristics of the aurora-A kinase and inhibitors in X-ray crystallized complex structures reported in the Research Collaboratory for Structural Bioinformatics (RCSB) protein databank (http://www.rcsb.org) [8]. The 19 aurora-A and ligand complex structures with potent activity (IC_50_ < 50 nM) were retrieved from the RCSB protein databank. We found that the 19 inhibitors used have a common scaffold in the form of an amine-based aromatic structure which includes quinazolines, imidazopyridines (3MYG [9]), imidazopyrazines (2X6D [10]), indazoles, pyrazoles (3FDN [3]), and substituted pyrimidines (2NP8 [11]). These amine moieties undergo a crucial H-bonding interaction with the residues around the A213 residue in aurora-A [2] and serve as criteria for filtering the numerous hits. These 19 structures were superimposed on a common reference (2NP8). We calculated the similarity between the 19 ligands extracted from the crystal structures using the Tanimoto coefficient by FCFP4 [12] with Pipeline Pilot 8.5 by Accelrys, Inc., as depicted in Table S1. Subsequently, cross-docking [13] studies of the 19 crystal structures were also performed using the LigandFit protocol [14], as shown in Table S2. Based on the results by the structural similarity levels and cross-dock scores, we finally selected five reference structures (2NP8, 2X6D, 3FDN, 3MYG, and 3P9J [15]) for the docking study.

**Figure 1:**
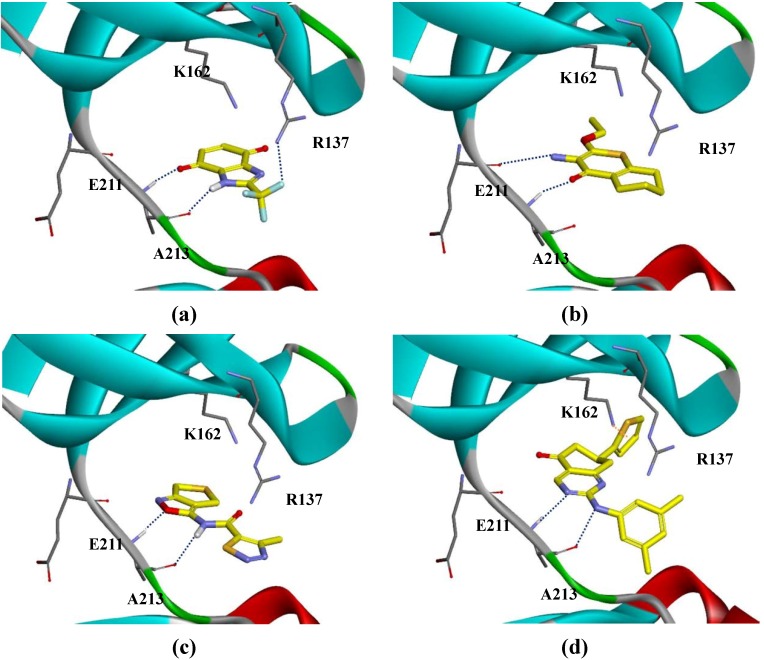
The binding mode of fragments: (**a**) 12; (**b**) 1; (**c**) 13; and (**d**) 3, (PDB code: 3P9J). Hydrogen bond is shown in blue dotted line and π-interaction is shown in orange dotted line.

After running the first docking protocol using the five reference structures, we identified 175 fragments which undergo crucial H-bonding interactions with the A213 residue. Finally, a more rigorous docking step produced 15 fragments showing greater amount of interaction, as mentioned in experimental section. Most of the fragments selected would dominantly interact by means of hydrogen bonding with A213, R137 and E211 and by π-bonding with K162 (see Figure 1).

In Table 1, we ascertained that fragment **12** had the highest ligand efficiency (4.14) [16] and that it could become a potential inhibitor.

**Table 1:** The dockscores and ligand efficiency for the selected 15 fragments.

Fragments	Dock Score					No. of Non-Hydrogen Atoms of the Fragments	Ligand Efficiency (LE) ^b^
	2NP8 ^a^	2X6D	3FDN	3MYG	3P9J		
**1**	− ^c^	−	48.05	−	−	15	3.20
**2**	−	40.70	−	−	33.82	24	1.55
**3**	31.00	35.70	33.85	34.41	37.35	25	1.38
**4**	50.33	−	46.34	51.84	55.99	20	2.56
**5**	−	−	31.55	−	31.42	15	2.10
**6**	−	−	−	−	32.59	18	1.81
**7**	53.69	−	48.64	54.98	53.74	21	2.51
**8**	−	−	28.83	−	27.84	15	1.89
**9**	−	−	−	−	28.95	16	1.81
**10**	−	−	35.45	3.76	10.23	19	0.87
**11**	51.44	−	44.62	36.03	30.65	21	1.94
**12**	−	−	−	−	62.06	15	4.14
**13**	−	−	13.95	−	24.95	17	1.14
**14**	15.59	57.47	37.42	15.34	49.61	23	1.53
**15**	55.56	54.99	55.43	57.07	55.17	22	2.53

^a^ Represented protein as a PDB code; ^b^ Ligand Efficiency (LE) = Mean of Dock Score/Non-hydrogen atoms of the fragments; and ^c^ A fragment does not bind to a protein.

### 2.2. The Biological Evaluation and Optimization

The 15 fragments obtained from the docking study were evaluated in terms of biological efficiency against the aurora kinase using the Kinase Profiler *in vitro* assay technique. This assay used the staurosporine (IC_50_ of 0.08 μM for aurora-A) [17] as a reference compound. The structures and biological activities of the 15 hit fragments and the reference compound are represented in Table 2. Among the suggested 15 fragments, we found that the most potent fragment was compound **12**, with 93% inhibition at a 100 μM concentration. In addition, three fragments (**1**, **3** and **13**) showed greater than 50% inhibition. This result supports our hypothesis that fragment **12** may be the best based on its docking score and ligand efficiency, as shown in Table 1. However, despite its good potency, fragment **12** was unmet on Lipinski’s rule for drug-likeness, resulting in poor physicochemical properties (consensus lipophilicity: 0.81, aqueous solubility: 2.87 mM). There was also a concern about the chemical stability of compounds containing the benzoquinone moiety. In an effort to improve these physical properties and chemical stability, we pursued a strategy to gain higher log*p* values as a descriptor for liphophilicity by coupling aryl groups, such as 6-phenylamine. Thus, Compounds **16** and **17** of the benzo[*d*]midazole-4,7-dione series as novel aurora-A inhibitors were derived from fragment **12** by referring to the work of Ryu *et al.* [18]. With this strategy, we found that the substituted benzoquinone analogues may have more or less reduced reactivity in the oxidation-reduction reaction [19]. In addition, it was observed that the *n*-halophenyl groups (see Table 3 and Figure S1) have higher lipophilicity (for ACD/log*p* values, **16** = 3.41, **17** = 3.56), resulting in a four-fold increase when compared to ACD/log*p* value of fragment **12**. The evaluation of the two compounds against aurora-family kinases demonstrated that they possessed good activity with not only aurora-A, but also with aurora-B. Precisely, compounds **16** and **17** showed inhibition level of 52% and 65%, respectively, at a concentration of 10 μM. These values are respectively equal to IC_50_ values of 9.17 and 7.47 μM for aurora-A kinase. They correspondingly showed 84% and 76% inhibition for aurora-B, values which are slightly better than those associated with of aurora-A, as shown in Table 3. However, this non-selectivity for aurora-A and aurora-B is not a problem. “As an example, **VX-680**, a potent inhibitor targeting both aurora-A and aurora-B kinases, has proceeded to clinical trials [1].

**Table 2:** The structure and effect of 15 fragments on Aurora-A inhibition.

Fragments	Structure	% Inhibition ^a^	Fragment	Structure	% Inhibition ^a^
**1**	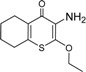	75 ± 1	**9**	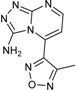	8 ± 10
**2**	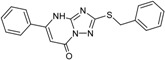	13 ± 12	**10**	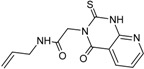	–
**3**	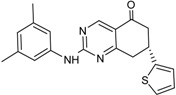	56 ± 6	**11**	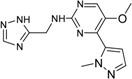	25 ± 2
**4**	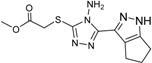	28 ± 1	**12**	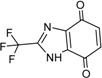	93 ± 2
**5**	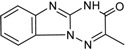	31 ± 6	**13**	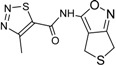	61 ± 2
**6**	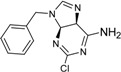	19 ± 2	**14**	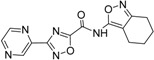	39 ± 3
**7**	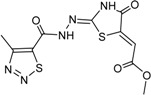	35 ± 3	**15**	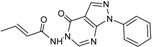	2 ± 15
**8**	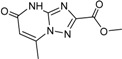	24 ± 4			

^a^ % inhibition at 100 μM concentration with standard deviation.

**Table 3:** The physicochemical properties, dockscores, and inhibition values of Aurora kinases by compound **16** and **17**.

Compounds	Structure	ACD/log*p* ^a^	Dock Score	IC_50_ (μM) ^c^	% Inhibition ^d^		
			3P9J ^b^		Aurora-A (h)	Aurora-B (h)	Aurora-C (h)
**16**	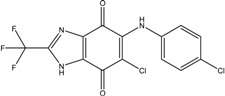	3.41	72.26	9.17	52	84 ± 1	8 ± 8
**17**	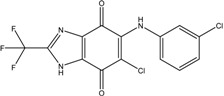	3.56	75.05	7.47	65	76 ± 1	17 ± 4
[17]	Staurosporine	4.4	81.53	0.08 (0.1) ^e^	100	–	–

^a^ p*K*a and ACD/log*p* calculated using the program ACD/Percepta 14.0.0 (Build 2203); ^b^ Represented protein as a PDB code; ^c^ At aurora A (h); ^d^ % inhibition at 10 μM; and ^e^ The value of parenthesis was obtained from reference paper [17].

**Figure 2:**
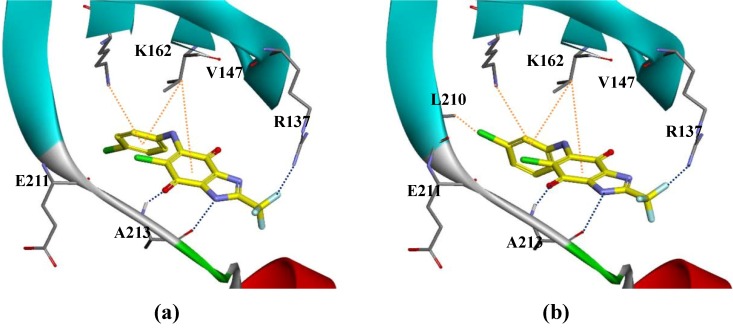
The binding mode of two compounds: (**a**) Compound **16**; and (**b**) Compound **17** (PDB code: 3P9J). Hydrogen bond is shown in blue dotted line and π-interaction is shown in orange dotted line.

In addition, the above-mentioned **CYC116** is currently undergoing Phase I clinical trials as an orally available aurora kinase inhibitor [5]. In contrast, no activity (**16** = 8%, **17** = 17%) was noted against aurora-C. Detailed data is provided in Tables S3–S6 and the binding modes of compounds **16** and **17** are depicted in Figure 2. Compound **16** and **17** were potently bound to the active site by three hydrogen bonds and two hydrophobic interactions, which reveal that the optimized compound with the *n*-Cl-phenyl group has extra interactions compared to fragment **12**. This docking result, along with the biological assay data, suggests that compounds **16** and **17** are potential inhibitors of aurora-A.

## 3. Experimental Section

### 3.1. The Filtering Process

We began with 4,340,181 compounds obtained from the well-known chemical database, eMolecules database (http://www.emolecules.com) [20]. Initially, we removed molecules including known reactive fragments or non-organic atoms. After this process, diverse fragment compounds having a molecular weight between 200 and 350 Dalton were continuously selected as representative molecules using a predefined set of properties; AlogP, the molecular weight, the number of hydrogen bond acceptors/donors, the Polar Surface Area, the number of rotatable bonds/aromatic rings, and functional class fingerprints (FCFP4), from Accelrys, Inc., DiscoveryStudio4.0 (San Diego, CA, USA).

### 3.2. Docking Study

Next, we carried out the docking study of the selected fragments using the released crystal complex structures of aurora-A from the RCSB protein databank. To consider the flexibility of the protein, we selected several complex structures for the docking study. Initially, aurora-A and ligand complex structures with potent activity (IC_50_ < 50 nM) were selected. These structures were superimposed on a common reference structure (2NP8). We calculated the degrees of similarity between the ligands extracted from the crystal structures using the Tanimoto coefficient by FCFP4 with Pipeline Pilot 8.5 by Accelrys, Inc. Subsequently, cross-docking studies of the selected crystal structures were also performed using the LigandFit protocol (4.0, Accelrys, San Diego, CA, USA). Based on the results of the structural similarity and the cross-docking scores, we finally selected the reference structures. Each protein was subjected to the in “Clean Protein” protocol and the CHARMm27 forcefield [21], after which cleaned protein was defined as a receptor molecule for the LigandFit docking calculation. The extracted ligands were fragmented according to two binding areas: The hinge region and another region. The fragments bound within 7 Å around the hinge region were used as Control Ligands in the LigandFit protocol. The hydrogen-bond acceptor (HBA) and hydrogen-bond donor (HBD) moieties in the hinge binding site were used as Interaction Filters in the LigandFit protocol. In order to prepare the input ligand molecules, energy minimization was conducted under the CHARMm27 force field; the conformations of the molecules were generated using the best conformation type. Each of the docked conformations was evaluated and ranked using the scoring functions including LigScore1, LigScore2 [22], Piecewise Linear Potential1 (PLP1), PLP2 [23], Jain [24], the Potential of Mean Force (PMF), PMF04 [25], the Ludi Energy Estimate (Ludi) 1, Ludi2, and Ludi3 [26].

Finally, a second docking run was performed on the previously selected compounds by following a more accurate procedure in which the interaction sites were added for each crystal structure. The following interaction sites were added for each protein: A213 –C=O as a hydrogen bond acceptor (HBA), A213 –NH as a hydrogen bond donor (HBD), P214 –C=O as a HBA, R137 –NH as a HBD, and K143 –NH as a HBD in the 2NP8 structure; A213 –C=O as a HBA and A213 –NH as a HBD in the 2X6D structure; A213 –C=O as a HBA, A213 –NH as a HBD, and E211 –C=O as a HBA in the 3FDN structure; A213 –C=O as a HBA and A213 –NH as a HBD in the 3MYG structure; and A213 –C=O as a HBA, A213 –NH as a HBD, E211 –C=O as a HBA, and G140 –CH_2_ as a hydrophobic site in the 3P9J structure.

### 3.3. Biological Assay

The fragments obtained from the above-mentioned docking study were purchased from commercial websites and evaluated using the Kinase Profiler *in vitro* assay technique developed by Merck Millipore, Inc., (Abingdon, UK). The aurora-A kinase was maintained with 8 mM myeloperoxidase (MPOS) at pH 7.0, 0.2 mM EDTA, 200 μM LRRASLG (Kemptide, American peptide Company, Sunnyvale, CA, USA), 10 mM MgAcetate, and [γ-33P-ATP]. The kinase reaction began with the addition of the MgATP mixture. The buffer-MgATP mixture was incubated for 40 min at room temperature. After incubation, the reaction was stopped through the addition of a 3% phosphoric acid solution. Then, a 10 μL reaction was spotted onto a P30 filtermat. The spotted P30 filtermat was washed three times for 5 min in 50 mM phosphoric acid and once in methanol prior to the drying and scintillation counting step. In addition, all physicochemical properties were estimated by ACD-Lab/Percepta software version 14.0.0 (Build 2203, ACD/Labs, Toronto, ON, Canada).

## 4. Conclusions

We analyzed the structural characteristics of the known aurora-A inhibitors using the Tanimoto coefficient and carried out a docking study of several protein structures to find a novel inhibitor against aurora-A. Our virtual screening model led to the discovery of new fragment which is the analogue of benzo[*d*]imidazole-4,7-dione. Based on this fragment, we found two compounds with potential inhibitory activity against aurora kinases, aurora-A and aurora-B.

## Supplementary Materials

Supplementary materials can be found at http://www.mdpi.com/1422-0067/15/11/20403/s1.
